# Linking Nanoscale Film Properties to Electrochemical Response in Cytochrome P450 Membrane Enzyme Films

**DOI:** 10.1021/acsmeasuresciau.5c00129

**Published:** 2025-11-18

**Authors:** Silan Bhandari, Matthew Dixon, Charuksha Walgama, Rajasekhara Nerimetla, Sadagopan Krishnan

**Affiliations:** † Department of Chemistry, 7618 Oklahoma State University, Stillwater, Oklahoma 74078, United States; ‡ 674961 Nanoscience Instruments, 10008 S. 51st Street, Suite 110, Phoenix, Arizona 85044, United States; § Department of Physical & Applied Sciences, 14842 University of Houston-Clear Lake, Houston, Texas 77058, United States

**Keywords:** nanoscale films, biomembranes, adsorption, surface modification, quartz crystal microbalance, electrochemical response

## Abstract

Membrane-bound human liver cytochrome P450 (CYP) enzymes catalyze key steps in xenobiotic metabolism, yet their integration into analytical devices depends critically on surface chemistry and film mechanics. Bacterial membrane preparations (“bactosomes”) provide abundant CYP sources for in vitro measurements and biosensors. Here, cystamine self-assembled monolayers (SAMs) on gold-coated quartz were compared with polyethylenimine (PEI)-coated gold for immobilizing CYP bactosomes. Quartz crystal microbalance with dissipation monitoring (QCM-D) tracked adsorption and film evolution, enabling quantitative estimates of the mass loading, thickness, and viscoelastic properties. After washing, bactosomes/SAM reached ∼113 nm thickness and 11.9 μg cm^–2^ mass, whereas bactosomes/PEI yielded ∼40 nm and 4.2 μg cm^–2^. Viscosity values were similar (2.5 cP), but elastic moduli differed: 2.0 × 10^4^ Pa for bactosomes/SAM vs 2.5 × 10^5^ Pa for bactosomes/PEI, indicating rigid films on PEI. Electrochemical characterization under argon and O_2_-saturated conditions showed higher faradaic responses for SAM-modified electrodes (*I*_p_ = 5.6 ± 0.6 μA in Ar; *I*_limiting_ = 95.9 ± 3.9 μA in O_2_) than PEI-coated electrodes (*I*_p_ = 3.2 ± 1.0 μA in Ar and *I*_limiting_ = 72.6 ± 3.3 μA in O_2_). These differences are attributed to the greater immobilized mass of bactosomes on the SAM-modified surface and the proportional electronic connectivity of their redox enzymes with the underlying gold electrode despite their higher film thickness. This work provides quantitative benchmarks for tuning film mechanics and coverage to optimize CYP-bactosome biocatalytic electrodes, informing the design of robust, high-signal platforms for biosensing and high-throughput drug metabolism assays. Correlation of QCM-D metrics with voltammetric outputs establishes a direct link among film mechanics, coverage, and electron-transfer efficiency, offering a generalizable framework for membrane enzyme immobilization. These measurements provide practical guidance for selecting surface chemistries when prioritizing either robustness or signal amplitude in CYP-based sensing architectures.

## Graphical abstract

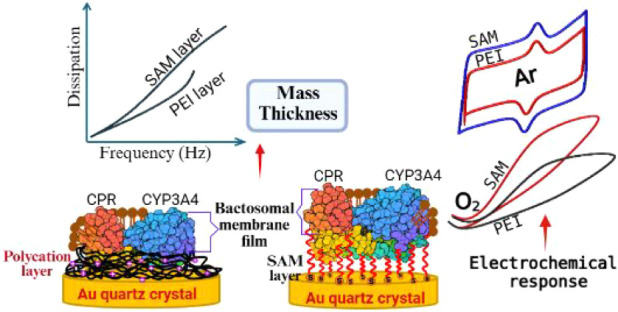

## 1 Introduction

The purification of membrane-bound enzymes is a tedious process for protein structure–function studies and for implementing useful enzyme catalytic properties in various large-scale synthetic and bioelectronics applications. In this perspective, the successful design of biofilms of membrane-bound major drug-metabolizing cytochrome P450 (CYP) enzymes is significant for the pharmaceutical industry and for the design of drug/pollutant biosensors and bioassays.^1–6^ CYP isolation and purification involve expensive, time-consuming procedures and yield only a very small amount of the purified enzyme. Hence, directly using the membrane-bound forms of the CYP enzymes present in liver microsomes, which are subcellular fractions obtained in vitro from the liver, or alternatively, CYP-expressed bacterial expression systems, is advantageous and has been documented in the literature.^6–8^

Notable successes in achieving electrocatalysis by purified liver CYP enzymes include layer-by-layer films with polyions on electrodes,^9^ CYP and NADPH–CYP reductase (CPR) fusion protein domains,^10^ and, more recently, nanomaterial-modified CYP bioelectrodes.^11^ However, designing biofilms of catalytically useful membrane-bound forms of CYP is still an expanding research area.^12^ Hence, bioelectrodes featuring direct membrane-bound fractions of CYP with its reductase partner, CPR, either coexpressed in bacteria (so-called bactosomes) or isolated from human and animal liver fractions (liver microsomes), have recently attracted increasing attention.^6,13,14^

Among analytical methods, quartz crystal microbalance with dissipation monitoring (QCM-D) is a sensitive analytical technique used to measure subtle changes in mass and viscoelastic properties at surfaces and interfaces in real time.^15–17^ QCM-D has gained considerable significance in the design and characterization of various nano and biointerfaces.^18–21^ Herein, we present QCM-D monitoring of CYP3A4 and CPR-containing bactosomal films upon adsorption onto a self-assembled monolayer or onto polycation (polyethylenimine) (PEI)-modified gold-coated quartz crystals (Figure 1A–B).

**Figure 1:**
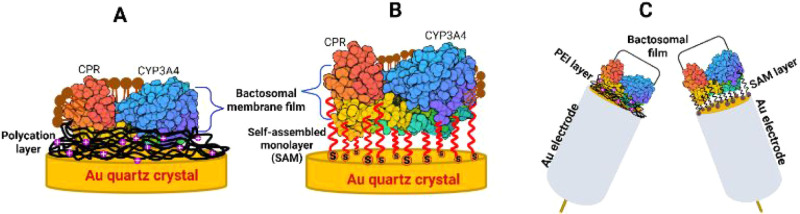
Schematics of adsorbed biofilms of membrane-containing bactosomal CYP3A4 + CPR onto **(A)** a polycation (polyethylenimine) layer coated on an Au quartz crystal or **(B)** a cystamine self-assembled monolayer on an Au quartz crystal, and **(C)** a polycation and a cystamine SAM layer assembled on a gold disk electrode surface.

Furthermore, we probed the cost-effective and efficient electrochemical approach^22,23^ and highlighted the direct electron transfer and electrocatalytic properties of CYP3A4 + CPR bactosomal film adsorbed separately on Au/cystamine and Au/PEI disk electrode surfaces (Figure 1C), mimicking the QCM-D gold quartz crystal surface. CYP3A4 is chosen because it is one of the major drug-metabolizing isoforms of CYP enzymes in the human liver.

The significance of our contribution in this article is related to both the combined methodology used and its application to a complex sample, membrane-bound CYP + CPR enzymes, which are relevant to pharmacology and toxicology. Real-time monitoring of a decrease in oscillation frequency (due to assembly formation) and an increase in dissipated energy (related to viscoelasticity) is achieved by using QCM-D. A physisorbed layer of a polycation (Figure 1A schematic) or a covalently modified SAM on gold-coated quartz crystal surfaces (Figure 1B schematic) is compared.

Results show that the membrane-bound CYP + CPR enzymes interact differently with cystamine SAM and PEI-deposited gold electrodes. The mass and nanometer-scale thicknesses of the modified surface layers, as well as those after adsorbing a bactosome layer, are presented. The peak currents and scan-rate-dependent peak potential shifts of the designed CYP3A4 + CPR bactosomal films were recorded. The anaerobic noncatalytic and electrocatalytic oxygen reduction peak current (*I*_p_) responses measured for Au/cystamine/CYP3A4 + CPR film were significantly higher than the peak current of Au/PEI/CYP3A4 + CPR bactosomal film, indicating the differences in the electrochemical properties of the polycation (PEI) and cystamine SAM modification on the Au electrode surface.

## 2 Experimental Section

### 2.1 Chemicals and Materials

Polyethylenimine (PEI) and cystamine (Sigma-Aldrich, St. Louis, MO, USA), CYP3A4 + CPR bactosomes with specific CYP concentration of 100 pmol mg protein^–1^ and CPR concentration of 190 pmol mg protein^–1^ (Cypex Ltd., Dundee, U.K.; U.S. distributor, Xenotech LLC), phosphate-buffered solution (PBS, 0.1 M, pH 7.4), gold electrode (geometric area, 0.125 cm^2^), potentiostat (CHI 6017 model), and a pH meter (Fisher Scientific, Model: AB15 Plus) were used.

### 2.2 QCM-D Monitoring

Solutions were pumped through the system at room temperature (23 °C) with a peristaltic pump. Two QCM-D sensors were tested simultaneously in the QCM-D flow module (Q-Sense Analyzer, Biolin Scientific, Inc.). The QSX 301 Gold sensors (14 mm diameter, AT-cut quartz crystals, nominal resonant frequency: 4.95 MHz, gold-coated, Biolin Scientific, Inc.) were cleaned in a UV/Ozone chamber for 5 min prior to use. First, the sensors were coated with a SAM of cystamine in ethanol or with a PEI aqueous solution at a flow rate of 200 μL/min. A QCM-D crystal was incubated with 10 mM cystamine in ethanol until a constant-frequency baseline was achieved. This was followed by a wash in phosphate-buffered saline, pH 7.4 (PBS), to remove any molecules, after which a new baseline at a lower frequency, indicative of an adsorbed layer, was observed. Similarly, a QCM-D crystal was coated with a PEI layer from a 2 mg/mL solution in deionized water. On both types of modified crystals, CPR-containing CYP3A4 bactosomes (2 mg/mL in PBS) were electrostatically adsorbed at a flow rate of 100 μL/min to determine the frequency decrease and the corresponding mass change. Additionally, the dissipation energy parameter was monitored at each step of the QCM-D surface modification.

### 2.3 Electrochemical Measurements

A three-electrode electrochemical cell was used, consisting of a gold (Au) disk electrode (diameter = 0.40 cm) as the working electrode, Ag/AgCl as the reference electrode, and a Pt wire as the counter electrode, all connected to a Gamry potentiostat for electrochemical measurements. The gold electrode surface was cleaned using an electrochemical cleaning protocol, beginning with an initial desorption scan in 0.5 M NaOH within a potential window of −0.8 to −1.4 V vs Ag/AgCl, followed by gold oxidation and reduction cyclic voltammetry (CV) scans in 0.05 M H_2_SO_4_ at a scan rate of 0.1 V s^–1^. This was followed by sonication of the electrodes in a 1:1 mixture of ethanol and 0.5 M aqueous NaOH for 10 min and then sonication in deionized (DI) water for 2 min.^24,25^

The cleaned gold electrodes were treated with 30 μL of either a cystamine self-assembled monolayer (SAM, 10 mM in ethanol) or a PEI solution (2 mg/mL in deionized water). The electrode was then placed in an ice-cold environment inside a closed box for 40 min, followed by rinsing with deionized water. Finally, 20 μL of a CPR-containing CYP3A4 bactosomal film was adsorbed onto the cystamine- or PEI-adsorbed gold electrode surface and incubated for an additional 40 min, followed by rinsing with DI water prior to electrochemical measurements, electrochemical impedance spectroscopy (EIS), and voltammetry.

For the EIS measurements, the electrodes were immersed in an aqueous solution containing 10 mM each of Fe­(CN)_6_^3–^ and Fe­(CN)_6_^4–^ in 0.1 M KCl, with the frequency scanned from 100 kHz to 0.5 Hz,10 mV of AC amplitude, and a 0.22 V applied DC potential vs a pseudo-Ag/AgCl reference electrode. The charge transfer resistance (*R*_ct_) was calculated using Gamry Echem Analyst software by fitting the Randles–Warburg circuit model in the Nyquist plot.

For the voltammetric measurements, the PBS solution (pH 7.4) was continuously purged with argon to determine direct electron transfer peaks between the bactosomal enzymes and the gold electrode or with oxygen for 30 min to determine the electrocatalytic reduction properties characteristic of heme enzymes present in bactosomes, such as P450 3A4, at room temperature (23 °C) before running the electrochemical measurements.

## 3 Results and Discussion

### 3.1 EIS Characterization

The EIS was applied to probe the surface features of the modified gold electrode and to identify the electrochemical performance.^26^ In EIS, an AC electrical signal over a range of frequencies is applied to observe the real (pure resistance) and imaginary (capacitive behavior) parts of the response. The real part consumes energy, while the imaginary part temporarily stores electrical energy and then returns it rather than using it up. The stored charge builds up at the interface between the electrode and the solution (like charging a microscopic capacitor). This is caused by delays in chemical reactions, such as oxidation and reduction, which catch up with the electrical signal. When we plot EIS data, the *x*-axis represents the real part of the impedance (resistance), and the *y*-axis represents the imaginary part (capacitive/reactive). When a system stores and releases electrical potential energy, an arc-shaped capacitance curve appears in the Nyquist plot rather than the straight-line characteristic of a pure resistor.

The arc corresponds to the semicircular region of the Nyquist plot, and its diameter indicates the magnitude of the charge-transfer resistance (*R*_ct_). In the EIS of a ferri/ferrocyanide redox system (a classic reversible electrochemical couple), the linear region that follows the semicircular arc at lower frequencies corresponds to diffusion-controlled behavior and is referred to as the Warburg impedance due to mass transport limitations, specifically, the diffusion of ferri- and ferrocyanide ions to and from the electrode surface.^27^

We scanned the EIS from 100 kHz to 0.5 Hz. The calculated *R*_ct_ values for the bare polished Au electrode, Au/PEI, and Au/PEI/CYP3A4 + CPR bactosomal film surface were 36, 48.8, and 98 Ω, respectively, as shown in Figure 2A. Similarly, the *R*_ct_ values for the Au/cystamine SAM layer and Au/cystamine/CYP3A4 + CPR bactosomal film surface were 94.6 and 349 Ω, respectively, as shown in Figure 2B. A significant difference in the charge-transfer resistance for the Au/PEI/bactosomal film (Δ*R*_ct_ = 62 Ω) and the Au/cystamine/bactosomal film (Δ*R*_ct_ = 313 Ω) was observed as compared to the bare gold electrode surface. The *R*_ct_ value increased on immobilization of bactosomal films on the modified gold electrode surface, which indicates that interfacial charge transfer was inhibited due to the adsorbed bactosomal film.^28^ This confirms the deposition of the bactosomal film layer on the modified gold electrode surface.

**Figure 2:**
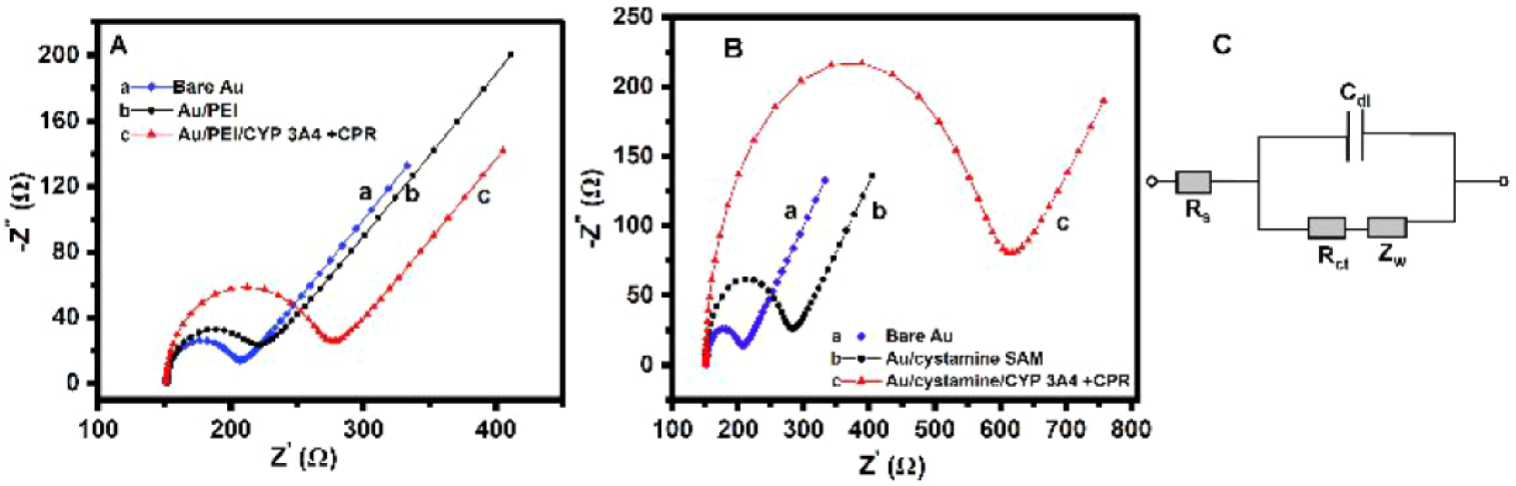
EIS characterization of the stepwise adsorption of CYP3A4 + CPR bactosomal film on a modified gold electrode surface. **(A)** Nyquist plot showing adsorption on Au/PEI-modified layer (curves **a, b**, and **c** labeled for bare Au, Au/PEI, and Au/PEI/CYP3A4 + CPR layer, respectively); **(B)** Nyquist plot showing adsorption on Au/cystamine SAM-modified layer (curves **a, b,** and **c** labeled for bare Au, Au/cystamine SAM layer, and Au/cystamine/CYP3A4 + CPR layer, respectively); **(C)** Randles circuit including the Warburg element (*R*_s_, *R*_ct_, *C*_dl_, and *Z*_w_ are the solution resistance, charge transfer resistance, electric double-layer capacitance, and Warburg impedance, respectively^29,30^ ).

### 3.2 Quartz Crystal Microbalance with Dissipation Monitoring (QCM-D)

Figure 3A and B show the QCM-D real-time data acquired for the formation of cystamine SAM and PEI coatings on the dual sensors, respectively, followed by the adsorption of CYP3A4 + CPR bactosomes onto each modified surface. The change in frequency (Δ*f*) corresponds to changes in mass (Δ*m*) per unit area of the quartz crystal; frequency decreases when mass is added to the surface of the sensor.^27,31^

**Figure 3:**
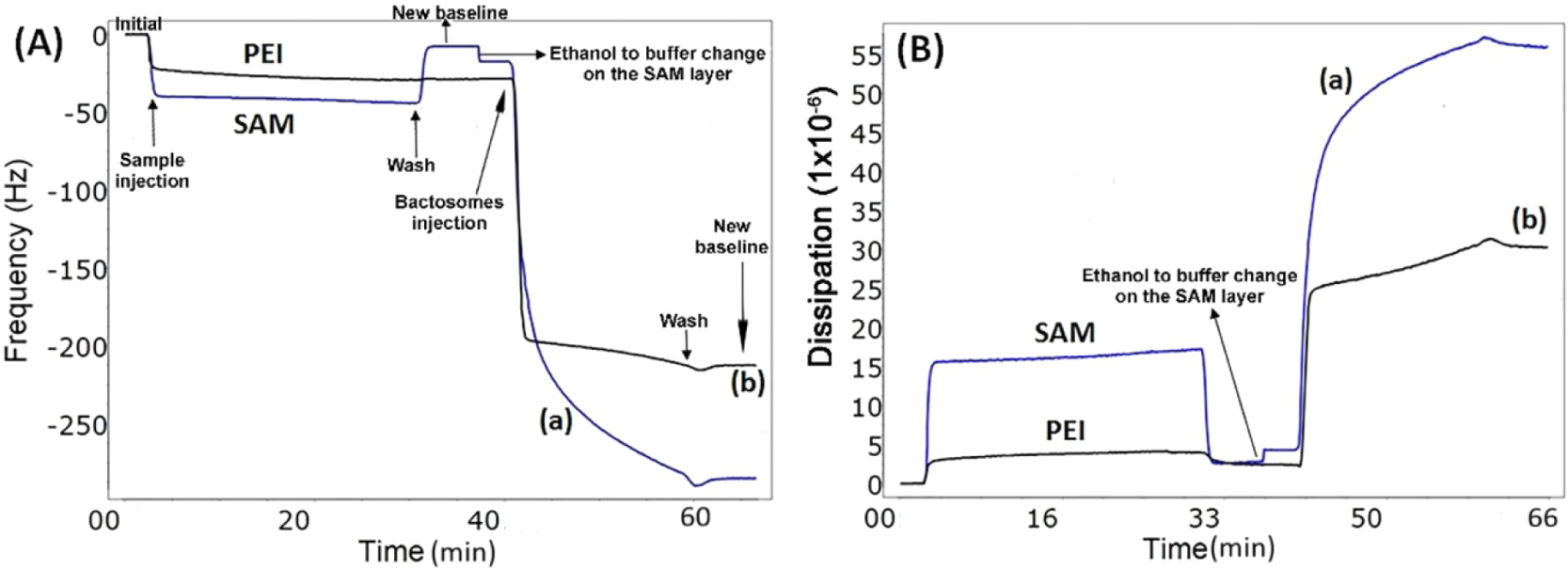
**(A)** Real-time decrease in frequency values (third harmonic) from the surface-adsorbed mass upon the formation of **(a)** SAM or **(b)** PEI-coated layers on QCM-D gold sensor crystals, followed by the adsorption of CYP3A4 + CPR bactosomes. **(B)** The corresponding increase in dissipation (third harmonic) values upon the formation of **(a)** SAM or **(b)** PEI-coated layers on QCM-D gold sensor crystals, followed by the adsorption of CYP3A4 + CPR bactosomes.

A change in dissipation (Δ*D*) corresponds to changes in viscoelasticity; when dissipation increases, the material on the sensor surface becomes softer. The plots of the third harmonic oscillation are presented in these figures. There appears to be a greater extent of bulk effects due to the changes in bulk liquid properties during SAM deposition (Figure 3A,B—curve a) than the PEI adsorption onto the gold crystals (Figure 3A,B—curve b). However, after rinsing the cystamine-incubated surface in ethanol or the PEI surface in PBS, the bulk effects are eliminated, and the thicknesses of the resulting layers can thus be reliably quantified. The shift in the baseline at ∼40 min for the SAM-modified crystal is due to the bulk effect of switching the solvent from ethanol to PBS for subsequent adsorption of bactosomes in solution.

The larger net signal changes for bactosomal deposition on the SAM surface compared to the PEI polycationic layer can be attributed to the following: (i) the amine groups of the self-assembled monolayers are arranged as oriented linear chains with solvent-exposed positively charged end surfaces and underlying accessible nonpolar ethylene groups, which favor better interaction with membranes of bactosomes upon adsorption; and (ii) interfacing between layers can additionally encapsulate more bactosomal membranes by creating microcavities than the relatively flat random-coiled PEI polymer bed, thereby limiting the surface loading of bactosomes beyond the electrostatic interactions.

Figure 4 shows the real-time change in dissipation with the oscillation frequency for the CYP3A4 + CPR bactosomal deposition onto a cystamine SAM- or PEI-modified sensor crystal. The dissipation vs frequency plot can help identify different “phases” during adsorption by revealing changes in slope. Here, the slope for bactosomal deposition is different on both SAM and PEI surfaces. This indicates that the bactosome adsorption behavior differs on each surface. The frequency axis can be thought of as the amount of added mass, and the dissipation axis can be thought of as representing the softness of the layer.

**Figure 4:**
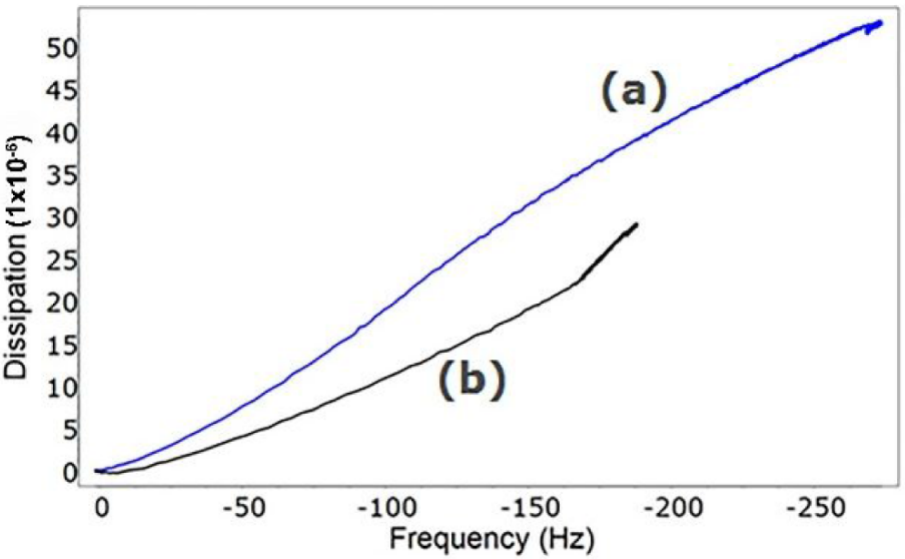
Dissipation vs frequency plots for **(a)** cystamine/bactosomal assembly and **(b)** PEI/bactosomal assembly.

Based on the Sauerbrey model, a linear relationship between Δ*f* and Δ*m* can be used to determine the adsorbed mass:

$$\Delta m=-C\frac{1}{n}\Delta f$$

$$C=\frac{t_{q}\rho_{q}}{f_{0}}$$

Where *n* is the harmonic number, *f*_0_ is the fundamental oscillation frequency of the crystal, *t*_q_ is the thickness of quartz, *ρ*_q_ is the density of quartz, and *C* is the mass sensitivity constant. *C* = 17.7 ng cm^–2^ Hz^−1^ for a crystal with a fundamental oscillation frequency of 5 MHz. Thus, for a mass change of 17.7 ng per square centimeter of geometric crystal area, a frequency change of 1 Hz results for a 5 MHz crystal. A precision of 0.01 Hz in a vacuum can be achieved with a QCM, enabling measurements of nanogram-scale masses. The film thickness is given by

$$\delta =\frac{\Delta m}{\rho }$$

Where *ρ* is the effective density of the adhering layer. Figure 5A shows the estimated mass and thickness of the sensor crystal after the cystamine SAM or PEI layer. Figure 5B displays the changes after adsorbing the CYP3A4 + CPR bactosomal film onto the modified surfaces.

**Figure 5:**
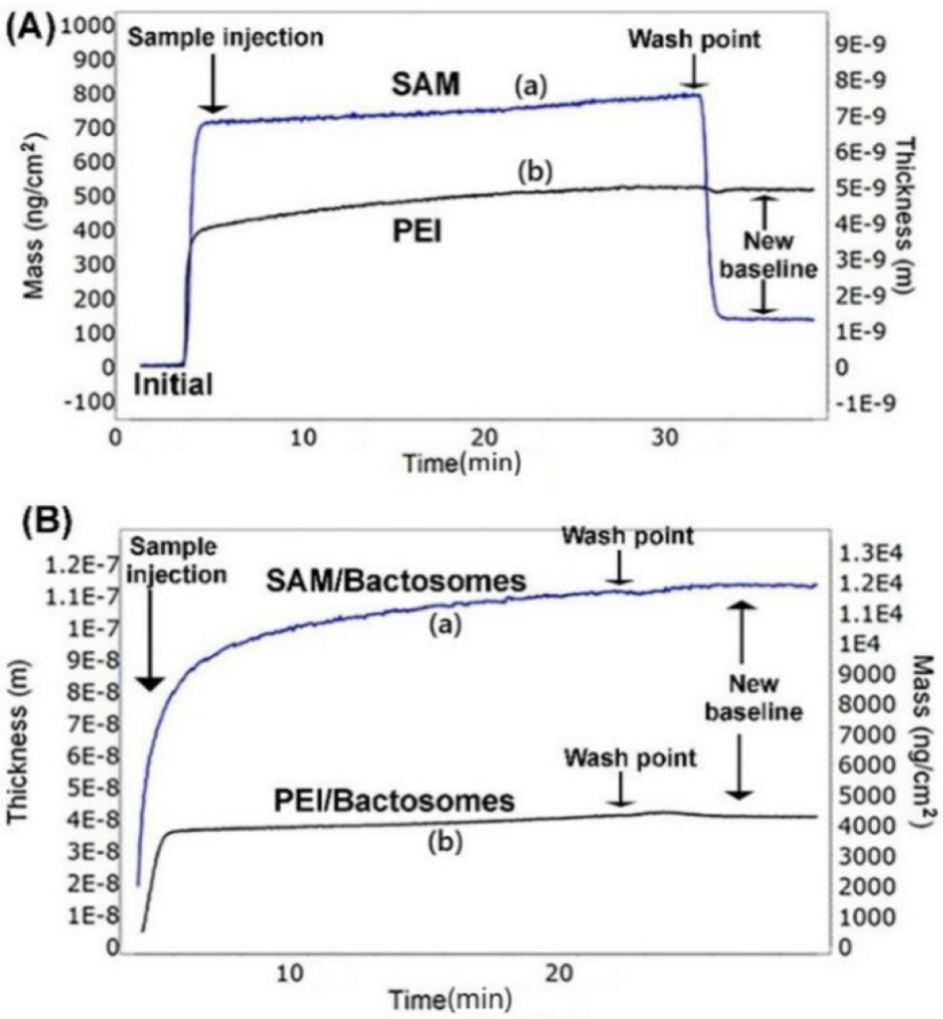
**(A)** Mass and thickness changes for **(a)** SAM or **(b)** PEI-coated layers on QCM-D gold sensor crystals. **(B)** Mass and thickness changes for CYP3A4 + CPR bactosomal adsorption onto **(a)** SAM or **(b)** PEI-coated layers on QCM-D gold sensor crystals.

Table 1 details the mass and thickness changes, along with the corresponding viscosity and elasticity values, for the designed cystamine SAM, PEI layers, and adsorbed bactosomal layers on the modified sensor crystals. Values at equilibrium before and after washing are presented. The thicknesses were calculated using a film density of 1050 kg/m^3^ for supported lipid bilayers, based on the viscoelastic Voigt model.^32,33^

**Table 1:** QCM-D Estimated Parameters for the Bactosomal Assemblies on Cystamine SAM- and PEI-Modified Crystals

Crystal modification	Thickness (nm)	Surface coverage (ng/cm^2^)	Viscosity (cP)	Elasticity (Pa)
At equilibrium before washing				
SAM	7.5	791	-	-
PEI	5.0	522	-	-
Bactosomes/SAM	111.2	11 677	2.5	2.33 × 10^4^
Bactosomes/PEI	41.0	4310	2.5	2.24 × 10^5^
At equilibrium after washing				
SAM	1.3	135	-	-
PEI	4.9	510	-	-
Bactosomes/SAM	112.9	11 857	2.5	2.02 × 10^4^
Bactosomes/PEI	40.4	4248	2.5	2.50 × 10^5^

The PEI layer is thicker than the cystamine monolayer after washing away the bulk of the unbound molecules. However, the bactosomal amount is about 3-fold higher on the monolayer surface than that on the PEI surface. As would be expected, the elasticity of the PEI polymer-modified surface with a bactosomal film is an order of magnitude greater than the nonpolymeric SAM-modified surface with an adsorbed bactosomal film.

### 3.3 Electrochemical Study of CYP Bactosomal Films

The direct electrochemical properties of CYP3A4 + CPR bactosomal films adsorbed on the Au/cystamine or Au/PEI layer were studied by using cyclic voltammetry. During cyclic voltammetry measurements (at a scan rate of 0.3 V s^–1^), Au/cystamine/CYP3A4 + CPR and Au/PEI/CYP3A4 + CPR bactosomal films showed a faradaic reduction peak at −0.40 ± 0.01 V and an oxidation peak at −0.34 ± 0.004 V, as shown in Figure 6A and B, respectively. The CVs of the bare Au electrode, Au/cystamine, and Au/PEI indicate no electroactive peaks, displaying only nonfaradaic charging current.

**Figure 6:**
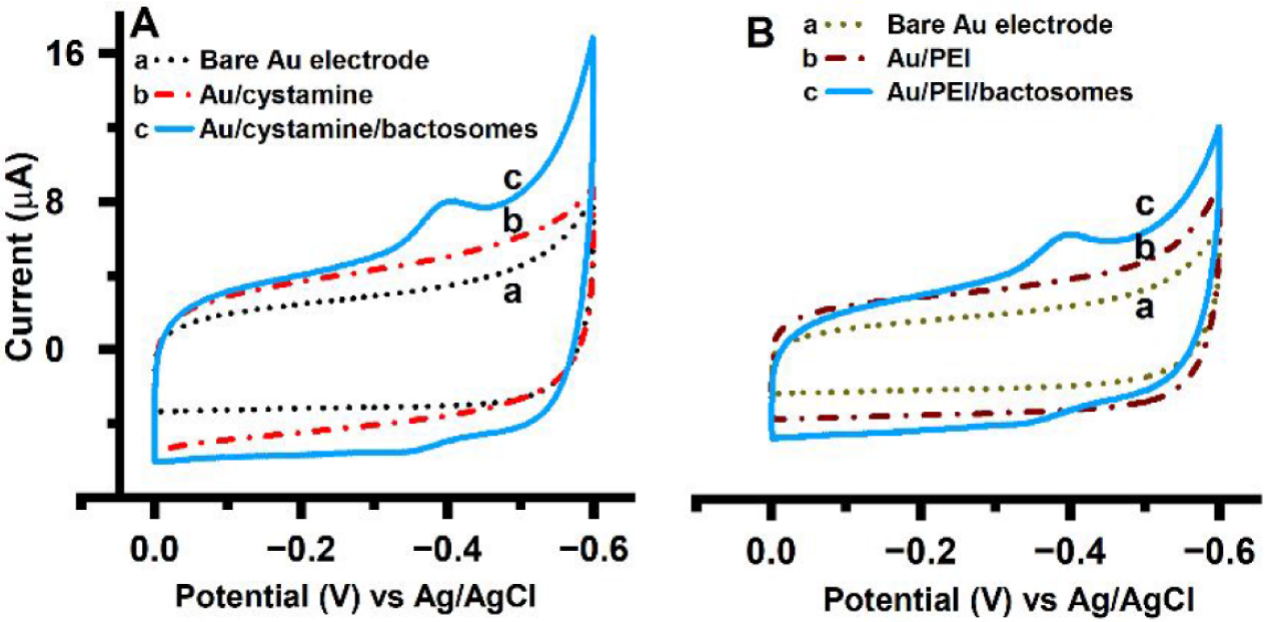
Cyclic voltammograms (*n* = 3) of **(A) (a)** bare Au electrode, **(b)** Au/cystamine, and **(c)** Au/cystamine/CYP3A4 + CPR and **(B) (a)** bare Au electrode, **(b)** Au/PEI, and **(c)** Au/PEI/CYP3A4 + CPR (conditions: argon-purged phosphate buffer solution, pH 7.4, 23 °C).

Prior studies showed that the CPR present in the CYP3A4 bactosomal film acts as an electron acceptor from the PEI- or SAM-modified gold electrode and transfers electrons to CYP3A4.^34,35^ When comparing CYP/CPR films to CYP alone, a significant shift of about 140 mV in CYP’s redox potential was observed, which was attributed to complex formation with CPR. The redox activity of CPR in CYP includes structural reorganization in addition to modifications in cofactor oxidation states. In particular, CPR_ox_ can undergo reduction within the CPR_ox_/CYP complex. The reduced form may quickly undergo structural reorganization to form a new complex in which CPR is reoxidized.^35^ The cause of the less prominent reverse peak in CYP3A4 + CPR bactosomal films adsorbed on Au/cystamine or Au/PEI layers, as shown in Figure 6A and B, which in turn leads to a diminished peak current and slows the efficiency of electron transfer in the reverse direction, requires further investigation in the future.

The CVs measured at 0.3 and 0.5 V s^–1^, which displayed faradaic peaks, were compared between the bactosomal film adsorbed on the Au/cystamine SAM layer and the Au/PEI layer to observe the reduction and oxidation peak responses, as shown in Figure 7A. The CVs of CPR-containing CYP3A4 bactosomal film on Au/cystamine displayed a greater faradaic peak response (*I*_p_ = 3.5 ± 0.5 μA at 0.3 V s^–1^ and *I*_p_ = 5.6 ± 0.6 μA at 0.5 V s^–1^) compared to the CVs observed on Au/PEI layer (*I*_p_ = 2.5 ± 0.6 μA at 0.3 V s^–1^ and *I*_p_ = 3.2 ± 1.0 μA at 0.5 V s^–1^), as shown in Figure 7A. The difference between the oxidation peak potential, *E*_p_(ox), and the reduction peak potential, *E*_p_(red), is represented by peak separation (Δ*E*_p_). In this study, the peak separation (Δ*E*_p_) was examined as the scan rate increased from 0.05 to 0.5 V s^–1^ to measure the rate of electron transfer during the redox interaction between the CYP3A4 + CPR bactosomal film and a PEI- or cystamine-coated gold electrode surface. The given equation can be used to determine the heterogeneous electron-transfer rate constant (*k*_s_) during the redox process between the bactosomal film and the PEI- and cystamine-coated Au surface using Laviron’s approach, which is based on Butler–Volmer surface-voltammetry theory.^35–37^

$$k_{s}=\frac{nFm\times \mathrm{scan rate}}{RT}$$

**Figure 7:**
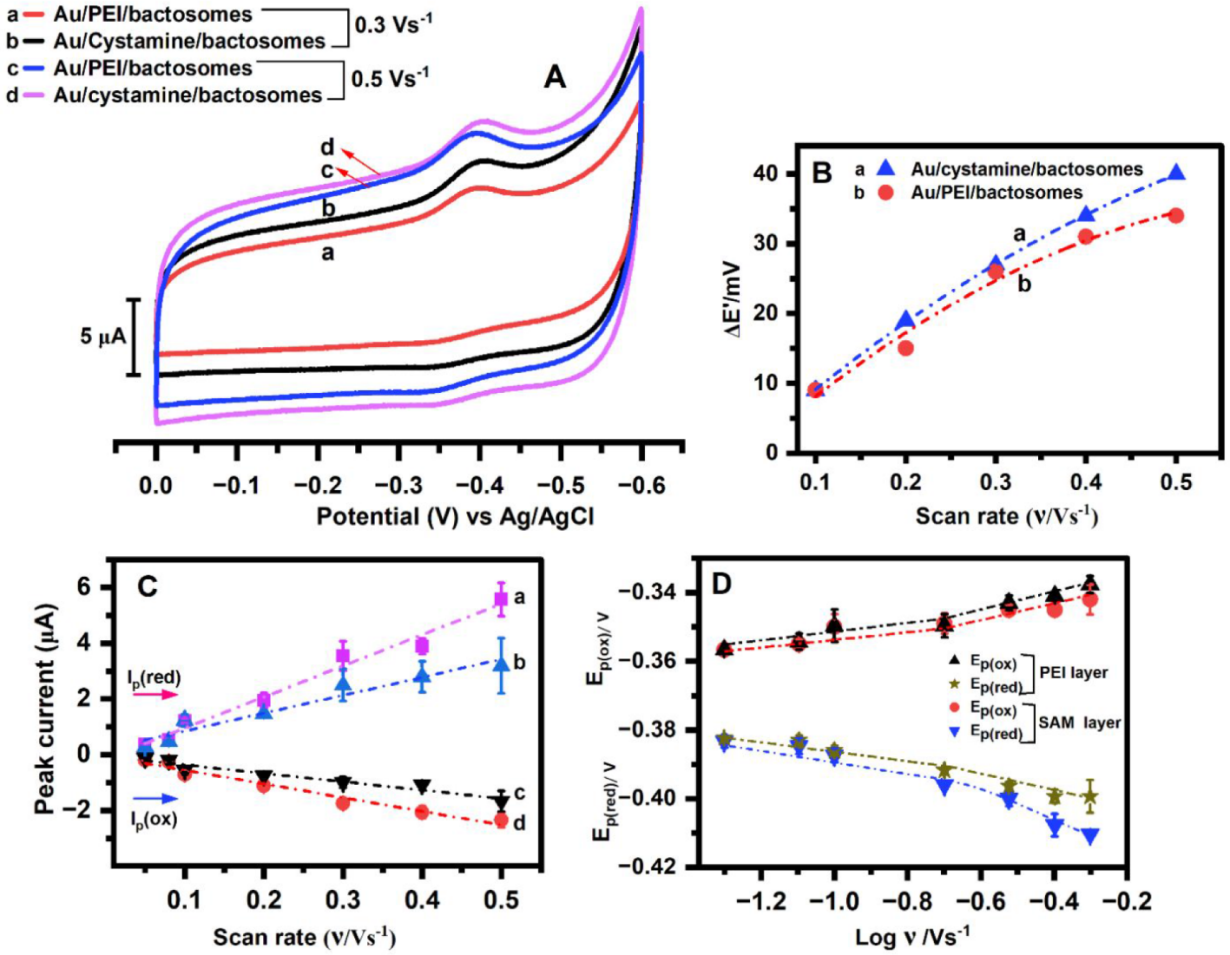
**(A)** A comparative plot of cyclic voltammograms (*n* = 3, measured at 0.3 and 0.5 V s^–1^) of Au/PEI/CYP3A4 + CPR (as **a** and **c**) and Au/cystamine/CYP3A4 + CPR (as **b** and **d** in the plot), bactosomal films displaying greater faradaic peaks on Au/cystamine SAM modification. **(B)** Experimental peak separation (corrected for the nonkinetic constant residual peak at low scan rates) with increasing scan rates for Au/cystamine/CYP3A4 + CPR (labeled as **a**) and Au/PEI/CYP3A4 + CPR (labeled as **b**) bactosomal films. **(C)** A plot of peak current (*I*_p_) vs scan rate (0.05 to 0.5 V s^–1^) showing greater peak current with Au/cystamine/CYP3A4 + CPR film (labeled as **a** and **d**), compared to that of Au/PEI/CYP3A4 + CPR film (labeled as **b** and **c**). **(D)** A plot of oxidation and reduction peak potentials vs the logarithm of the scan rate for the Au/cystamine/CYP3A4 + CPR bactosomal film and the Au/PEI/CYP3A4 + CPR film. Conditions were argon-purged PBS buffer, pH 7.4, and 23 °C.

Where *R* is the gas constant (8.314 J mol^–1^ K^–1^), *F* is Faraday’s constant (96 485 C mol^–1^), and *n* is the number of electrons participating in the electron transfer. *T* is the temperature in Kelvin, and according to Laviron, *m* is a variable with a nonlinear inverse relationship to the observed peak separation (*m* ∝ 1/*n*Δ*E*_p_).^36–38^ Consequently, the peak separation between *E*_p_(red) and *E*_p_(ox) is minimal for a reversible redox process where rapid reduction and oxidation processes occur, where *m* increases, and where *k*_s_ is higher. Conversely, when a protein film on an electrode surface undergoes a slow or quasi-reversible redox process, *k*_s_ is moderate to low, and the peak separation increases with increasing scan rate.^4,35,39^

The nearly constant peak separation values at low scan rates (<0.1 V s^–1^) were averaged and then subtracted from the linearly increasing peak separation values with respect to scan rate (0.1 to 0.5 V s^–1^) in order to calculate *k*_s_. The plot of corrected experimental peak separation vs scan rate (0.1 to 0.5 V s^–1^) for Au/cystamine/CYP3A4 + CPR and Au/PEI/CYP3A4 + CPR bactosomal films is shown in Figure 7B. The calculated *k*_s_ value for CYP3A4 + CPR bactosomal film adsorbed on the Au/cystamine and Au/PEI surfaces was 16.2 ± 0.5 and 18.9 ± 1.5 s^–1^, respectively, as shown in Table 2.

**Table 2:** Electrochemical Properties of the Bactosomal Film on Modified Au Surface (Phosphate Buffer, pH 7.4, *n* = 3)

Bactosomal film on the Au electrode surface	Apparent surface coverage, Γ (pmol/cm^2^) (average ± SD)	PWHM/mV (average ± SD)	Formal potential (*E*°^′^/V) (average ± SD)	*k*_s_ (s^–1^) (average ± SD)
Cystamine SAM/CYP3A4 + CPR	14.0 ± 1.4	69 ± 3	–0.373 ± 0.002	16.2 ± 0.5
PEI/CYP3A4 + CPR	10.9 ± 1.1	70 ± 4	–0.369 ± 0.001	18.9 ± 1.5

By utilizing CVs, the previous study reported formal potential (*E*°′ vs Ag/AgCl, PBS pH 7.4) and heterogeneous electron transfer rate constants (*k*_s_) for CPR (*E*°′ = −0.427 V and average *k*_s_ ≈ 42 s^–1^) and CYPs + CPR (*E*°′ = −0.447 V vs Ag/AgCl and average *k*_s_ ≈ 40 s^–1^) layer-by-layer films electrostatically adsorbed on PG/PDDA/PSS electrodes (where, PG = Pyrolytic graphite, PDDA = poly­(diallyldimethylammonium chloride), and PSS = poly­(sodium 4-styrenesulfonate).^35^ Furthermore, the *k*_s_ reported for PG/PEI/PSS/P450 2E1 film was 18 ± 4 s^–1^ at pH 7.^39^ Moreover, the electron transfer rates reported for P450 2E1 (PBS pH 7.0) adsorbed on Au/cystamine/maleimide layer, Au/MPA/PDDA layer (MPA = mercaptopropionic acid), and bare glassy carbon (GC) electrode were 10 ± 0.5 s^–1^, 2 ± 0.5 s^–1^, and 5 ± 0.5 s^–1^, respectively.^40^ This suggests that our calculated *k*_s_ value for the CYP3A4 + CPR bactosomal film, *k*_s_ = 16.2 ± 0.5 s^–1^ on the Au/cystamine layer and *k*_s_ = 18.9 ± 1.5 s^–1^ on the Au/PEI layer-modified gold electrode surface, falls within a similar range to previously reported values.

To study redox kinetics at the bactosomal film–Au electrode interface, the effects of the scan rate on peak current (*I*_p_) and peak potential (*E*_p_) were examined. The plot of the peak current vs scan rate, from 0.05 to 0.5 V s^–1^, was linear for CYP3A4 + CPR bactosomal film adsorbed on an Au/cystamine or an Au/PEI surface layer, as shown in Figure 7C. The linear regression equations for cathodic and anodic peaks were measured for Au/cystamine/CYP3A4 + CPR film as *I*_pc_= 11.14υ – 0.15 (*R* ^2^ = 0.97) and *I*_pa_= −4.93υ – 0.05 (*R* ^2^ = 0.96), respectively. Similarly, the equations obtained for Au/PEI/CYP3A4 + CPR bactosomal film were *I*_pc_= 6.43υ + 0.20 (*R* ^2^ = 0.94) for the cathodic peak and *I*_pa_= −3.019υ – 0.06 (*R* ^2^ = 0.93) for the anodic peak. The peak potential (*E*_p_) shift measured with the logarithm of scan rates (0.05 to 0.5 V s^–1^) for the Au/cystamine/CYP3A4 + CPR bactosomal film is shown in Figure 7D. A similar *E*_P_ shift was also measured for Au/PEI/CYP3A4 + CPR bactosomal films across the same scan rate (0.05 to 0.5 V s^–1^) as shown in Figure 7D.

The electroactive amount (Γ) of CYP3A4 + CPR bactosomal film adsorbed on the Au/cystamine SAM layer was greater than that of Au/PEI/CYP3A4 + CPR film (Table 2) in accordance with the respective higher mass surface coverage and the associated film thickness (Table 1), thus offering complementary microstructural film insights. The mass of the bactosomal film per unit area of the Au-coated quartz crystal (surface coverage), as measured during the QCM-D experiment, reflects the total mass of the film, which includes both redox-active proteins (CYP3A4 and CPR) and nonredox-active components such as phospholipid membranes, other structural proteins, and polymers. In contrast, in the CV experiment, the surface coverage determined by integrating the charge (*Q*) from the faradaic redox peak corresponds exclusively to the electroactive bactosomal redox species.

The average formal potential (*E*°′) observed for Au/cystamine/CYP3A4 + CPR was around −0.373 ± 0.002 V vs Ag/AgCl, which is similar to the formal potential for the Au/PEI/CYP3A4 + CPR film (−0.369 ± 0.001), as shown in Table 2. The measured formal potentials (*E*°′) were in good agreement with previously reported values. Specifically, CYP3A4/CPR microsomes immobilized on a naphthalenethiolate (NT)-modified gold electrode exhibited a formal potential of −0.40 V vs Ag/AgCl at pH 7.4.^41^ To achieve a more precise understanding, electrochemical measurements were performed on only CYP3A4 (without CPR) and only CPR microsomes (without CYP), which displayed distinct redox couples with formal potentials of −0.38 V for CYP microsomes and −0.41 V vs Ag/AgCl for CPR microsomes.^41^ The formal potential of −0.44 ± 0.04 V vs Ag/AgCl was also reported for the CYP3A4 + CPR bactosomal film adsorbed on Au/cysteamine surface.^14^ Moreover, a formal potential of −0.41 V vs SHE has also been determined by redox titration of CYP3A4 bound to lipids.^42^ Furthermore, cytochrome P450 incorporated into polyion layer-by-layer (LbL) films on pyrolytic graphite electrodes showed a formal potential of approximately −0.35 V vs SCE at pH 7.0 (Table 3).^39,43^

**Table 3:** Comparison of Formal Potential (*E*°′) of the CYPs Adsorbed on the Modified Electrode Surface under Anaerobic Phosphate Buffer Solution

Bactosomal film	Electrode modification	*E*°′	ref(s)
CYP3A4 + CPR	Au/naphthalenethiolate (NT)	–0.40 V vs Ag/AgCl	^41^
CYP-microsomes (without CPR)	Au/NT	–0.38 V vs Ag/AgCl	^41^
CPR-microsomes (without CYP)	Au/NT	–0.41 V vs Ag/AgCl	^41^
CYP3A4 + CPR	Au/cysteamine	–0.44 V vs Ag/AgCl	^14^
CYP3A4 bound to lipids	CYP3A4-Nanodisc	–0.41 V vs SHE	^42^
CYP	PG/polyion layer-by-layer (LbL)	–0.35 V vs SCE	^39,43^
CYP3A4 + CPR	Au/PEI and Au/cystamine SAM layer	–0.37 V vs Ag/AgCl	This work

The peak widths at half-maximum (PWHM) for the bactosomal film adsorbed on the Au/cystamine layer and Au/PEI layer were 69 ± 3 and 70 ± 4 mV, respectively, as shown in Table 2. CPR acts as an electron donor by using flavin mononucleotide (FMN) and flavin adenine dinucleotide (FAD) as electron transfer cofactors during cytochrome P450-catalyzed monooxygenase processes. One FAD and one FMN molecule are present in CPR, which transfer electrons from NADPH to the heme iron of CYPs, one at a time. The entry and departure points for these molecules are FAD and FMN, respectively. At catalytic turnover, the one-electron-reduced semiquinone of FMN is at its highest oxidation state, and the enzyme alternates between 1e and 3e reduced levels (or 2e and 4e).^34,35^ In our study, the observed PWHM values for the bactosomal film (CYP3A4 + CPR) were lower than the theoretical 90.6 mV expected for a one-electron transfer in an ideal film of an adsorbed species on the electrode surface. This suggests an intermediate, mixed-electron-transfer process with a value >1.0, potentially involving the association of CYP and CPR as a complex within the membrane, and a redox path in CPR involving FMN and FAD oxidation–reduction.^34,35^ A number of variables, including the orientation, chemical structures, and redox potentials of the electroactive cofactors FMN and FAD in the enzyme, affect the direct electron transfer between the CPR and acceptor proteins. Moreover, the peak current ratio (*I*_pa_/*I*_pc_) calculated for CYP3A4 + CPR bactosomal films adsorbed on the Au/cystamine layer or Au/PEI layer was less than 1.0, as the reverse peak intensity was significantly diminished, likely due to CPR/CYP complexation and structural reorganization. These findings offer insights into the feasible enzyme-reductase complexes in the immobilized membrane fraction and the resulting observed electrochemical properties.^5,44^

### 3.4 Catalytic Oxygen Reduction Properties of Bactosomal Film

The oxygen reduction electrocatalytic behavior of the bactosomal film adsorbed on the Au/cystamine SAM layer and the Au/PEI layer was studied using the cyclic voltammetry measurements under a saturated oxygen buffer solution (pH 7.4) at 23 °C. The oxygen reduction voltammograms of the Au/cystamine/CYP3A4 + CPR bactosomal film, the Au/cystamine layer, and the bare Au electrode surface are shown in Figure 8A (labeled as a, b, and c, respectively). Similarly, Figure 8B represents CVs of Au/PEI/CYP3A4 + CPR, the Au/PEI layer, and bare Au (labeled as a, b, and c, respectively). The Au/cystamine/CYP3A4 + CPR bactosomal film shows a greater oxygen reduction current (*I*_p_ = 95.9 ± 3.9 μA at 0.3 V s^–1^) compared to the Au/PEI/CYP3A4 + CPR bactosomal film (*I*_p_= 72.6 ± 3.3 μA at 0.3 V s^–1^), which further indicates the greater adsorption and electrocatalytic properties of the bactosomal film adsorbed onto the Au/cystamine SAM layer.^14^

**Figure 8:**
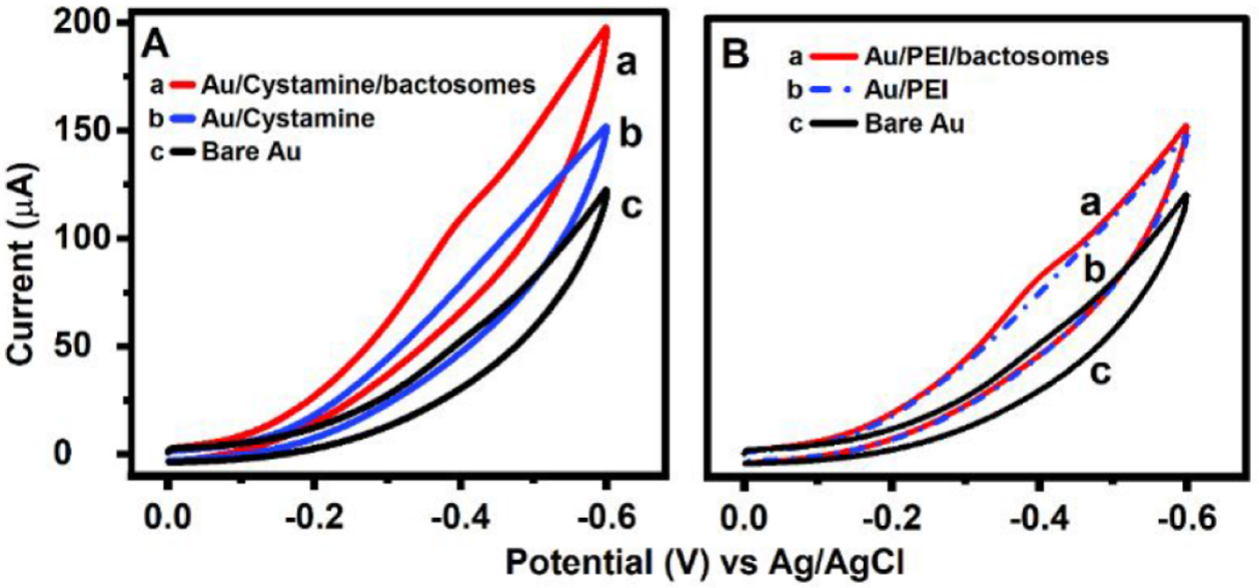
Electrocatalytic oxygen reduction voltammograms of **(A) (a)** Au/cystamine/CYP3A4 + CPR bactosomal film, **(b)** Au/cystamine layer, and **(c)** bare Au, and **(B) (a)** Au/PEI/CYP3A4 + CPR bactosomal film, **(b)** Au/PEI layer, and **(c)** bare Au in a saturated oxygen-purged phosphate buffer (pH 7.4) solution at 23 °C.

Direct electrochemistry is CYP-reductase-mediated, so the total electroactive amount does not fully reflect the total amount of P450, as observed in isolated purified P450 bioelectrodes. Hence, the observed limiting current does not directly relate to the P450 electroactive amounts. Furthermore, full electrocatalytic activity was observed only on the bactosomal-adsorbed layer, not from the polymer-only layer (Figure 8B). This shows that the distance from the electrode is a key factor influencing the electroactive behavior of the protein film.^45^ As the PEI layer is relatively thick and coiled compared to the SAM layer, it could increase the distance between the Au surface and the redox-active heme center in the bactosomal film.

The cystamine SAM layer is a highly ordered linear chain on the gold electrode surface, which facilitates greater adsorption of redox-active bactosomal film through solvent-exposed groups (both nonpolar methylene tail and polar amine end groups) for hydrophobic and electrostatic interactions. A possible interlayer gap among the SAM chains enables more encapsulation of bactosomes compared to the coiled flat PEI layer. The above-described SAM layer features thus overcome the higher charge-transfer resistance (Figure 2B) due to more adsorbed mass and their electronic connectivity with the electrode. Therefore, the insulating barrier caused by the higher adsorbed mass of bactosomal film on the SAM-coated Au surface contributes to the higher *R*_ct_.^46–48^ However, the heterogeneous electron-transfer kinetics (*k*_s_) are slightly lower than or similar to those of the PEI-adsorbed CYP3A4 + CPR film. The rate constant (*k*_s_) of a long-range electron transfer reaction exhibits an exponential dependence on the donor–acceptor distance (*d*), as described by the equation, *k*_s_ ∝ exp­(−β*d*),^49–51^ where *d* is the distance between the electrode surface and the enzyme’s redox-active site, and β is the decay coefficient, which depends on the medium the electron tunnels through. As the electron transfer constant (*k*_s_) exhibits an exponential decay with an increasing distance between the gold electrode surface and the redox center of the bactosomal film (CYP3A4 + CPR), the nanometer-scale thickness of the cystamine SAM layer is less than that of the PEI layer. Thus, the thinner, highly ordered linear chain of cystamine SAM permits electron tunneling between the gold electrode and the redox-active P450 bactosomal film, resulting in a similar response in the *k*_s_ value to that of the thicker PEI layer system, despite the high R_ct_ in the SAM layer-adsorbed bactosomal mass. Hence, more molecules of bactosomal redox proteins are connected to the SAM-modified electrode, yielding higher electroactive protein amounts and catalytic currents than the PEI-modified bactosomal surface.

Physical adsorption of enzymes onto bare or modified electrodes is a simple immobilization technique that generally preserves enzymatic activity; however, it is limited by poor stability.^52,53^ A prior study reported that an Au/cysteamine/P450 3A4 + CPR bactosomal film on gold electrodes exhibited a half-life of 4 h based on amperometric *i*–*t* analysis.^14^ Another investigation evaluated the electrochemical stability of human liver microsomal (HLM) films containing CYP enzymes with CPR reductase adsorbed on glassy carbon (GC), basal-plane pyrolytic graphite (BPG), and high-purity graphite (HPG) electrodes. After 200 continuous CV cycles over 40 min in anaerobic pH 7.0 buffer, the retained current was approximately 30% for GC/HLM, 50% for BPG/HLM, and 85% for HPG/HLM films.^3^ Additionally, a separate study showed that liver microsomal bioelectrodes maintained good stability, with less than a 15% decrease in relative impedance over 40 h.^5^ Collectively, these findings suggest that bactosomal films adsorbed onto PEI- or cystamine–SAM-modified gold electrodes can be used effectively for electrocatalytic and biosensing applications. However, issues such as film loss and reductive thiol desorption from gold surfaces limit their use to single-use applications due to weak physisorption.^14^

### 3.5 Frequency-Dependent Study of CYP Bactosomal Film

Square-wave voltammetry was used to study frequency-dependent voltammograms (ranging from 5 to 25 Hz) for a CYP3A4 + CPR bactosomal film adsorbed on a cystamine SAM or a PEI-coated Au electrode surface layer under oxygen and argon conditions, as shown in Figure 9A and B. The voltammograms in an oxygen environment showed a higher peak current than that in argon, revealing the electrocatalytic properties of the heme center in the CYP3A4 enzyme under oxygen conditions (Figure 9A–C). The Au/cystamine/CYP3A4 + CPR film exhibits a higher peak current response, *I*_p_ = 25.8 ± 3.4 μA, as compared to that of the Au/PEI/CYP3A4 + CPR film response, *I*_p_ = 18.5 ± 0.5 μA, at 25 Hz under oxygen conditions, as shown in Figure 9C. This further confirmed the results obtained from the QCM-D sensor crystals and cyclic voltammetry studies. Moreover, the peak shifts with increasing frequency (5 to 25 Hz) were studied for the CYP3A4 + CPR bactosomal film under an oxygen environment. The CYP3A4 + CPR film adsorbed on the Au/cystamine layer and the Au/PEI layer exhibited a similar peak shift (between 5 and 25 Hz), Δ*E*_p_ = 20.3 ± 4.8 mV, and Δ*E*_p_ = 21.3 ± 4.0 mV, respectively, as shown in Figure 9D, indicating a similar rate.

**Figure 9:**
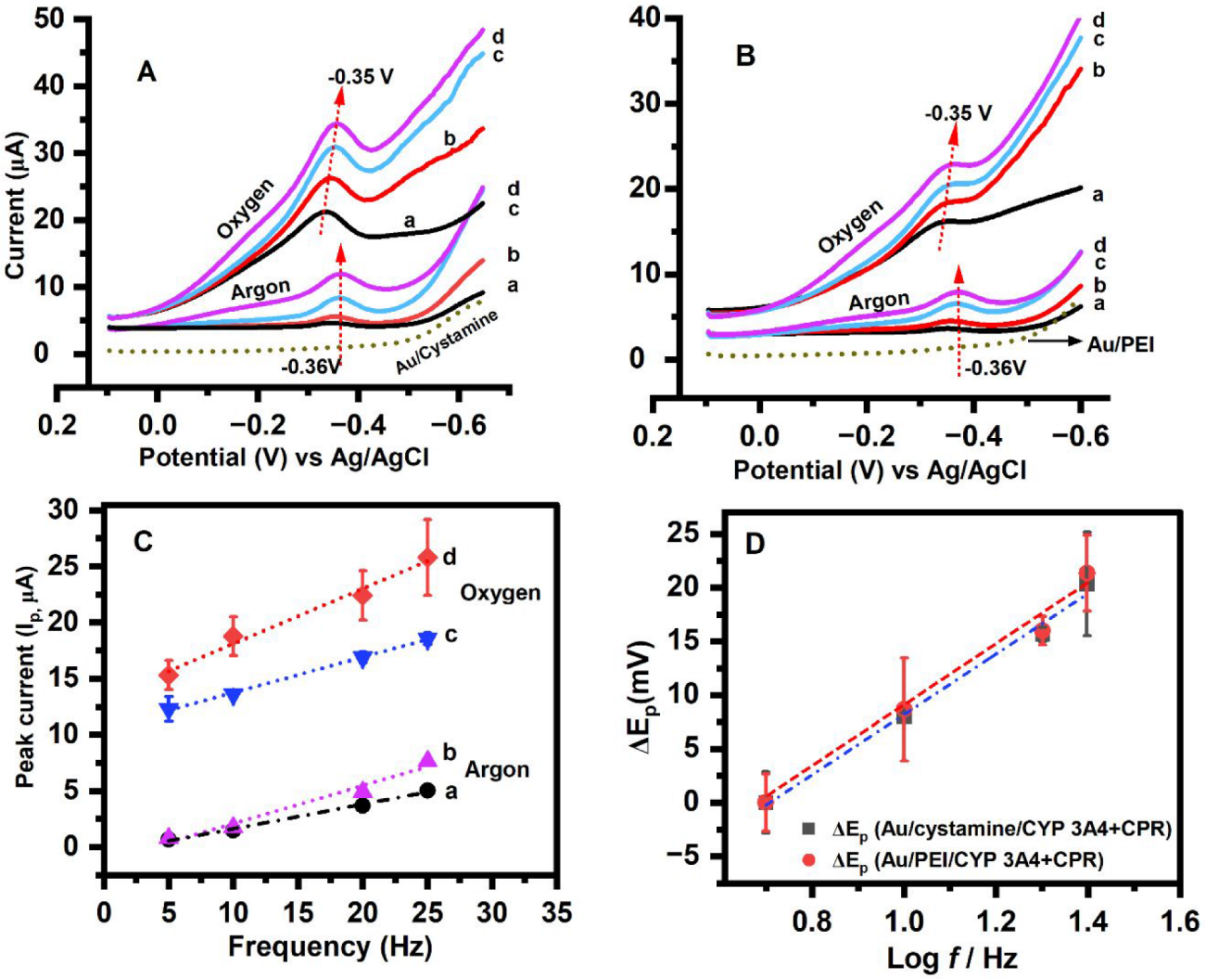
Comparative study of sweep frequency dependence on square wave voltammograms (5, 10, 20, and 25 Hz labeled as **a, b, c**, and **d**, respectively) by purging oxygen and argon in pH 7.4 phosphate buffer solution (PBS) at room temperature (23 °C). **(A)** Au/cystamine/CYP3A4 + CPR bactosomal film voltammograms. **(B)** Au/PEI/CYP3A4 + CPR bactosomal film voltammograms. The oxygen-purged condition shows a greater faradaic peak current than the anaerobic (argon) condition at increasing frequencies from 5 to 25 Hz, as shown in the voltammograms in **(A)** and **(B)**. **(C)** The peak current (*I*_p_) vs frequency plot shows a higher increase in peak current under oxygen conditions (**c, d**) as compared to that under argon conditions (**a, b**) with respect to increasing frequency. The peak current (*I*_p_) is greater in the case of Au/cystamine/CYP3A4 + CPR film (represented as **b** and **d**) when compared to Au/PEI/CYP3A4 + CPR bactosomal film (represented as **a** and **c**) under both argon and oxygen conditions, respectively. **(D)** A plot of peak shift (in mV) vs logarithm of frequency (log_10_ *f*) of Au/cystamine/CYP3A4 + CPR film and Au/PEI/CYP3A4 + CPR film under oxygen conditions shows a similar peak potential shift. (The square wave voltammetry parameters used were, potential range from 0.1 to −0.65 V with a 4 mV step height and a 25 mV pulse height.)

The electron transfer is likely due to the same type of electron-receiving CPR molecule in the bactosomes.^54,55^ In contrast, they are adsorbed in greater quantities on the SAM than on the PEI-modified surface.

## 4 Conclusions

Deposition of cystamine and polyethylenimine (PEI) onto Au-coated QCM-D crystals was monitored, yielding layer thicknesses of ∼1 nm (cystamine SAM) and ∼5 nm (PEI). Quantitative QCM-D analysis showed that, despite its thinner primer thickness, the cystamine SAM supported markedly greater bactosome adsorption, producing thicker, more massive films than PEI. We attribute this outcome to differences in interfacial chemistry and steric environment: the amine-terminated SAM likely presents favorable electrostatic interactions and lower steric hindrance for vesicle attachment, whereas the coiled PEI layer forms a denser polymer cushion that limits loading, consistent with the observed higher film stiffness on PEI. Electrochemical measurements further demonstrated higher faradaic currents for Au/cystamine/CYP3A4 + CPR electrodes than for Au/PEI/CYP3A4 + CPR, consistent with greater immobilized mass and more effective electronic coupling to the underlying gold, despite the higher overall film thickness. Together, these results establish a practical design rule: PEI yields thinner, more rigid films, while cystamine SAMs deliver a higher enzyme coverage and stronger electrochemical signals. Future work will profile CYP activity toward drug candidates in these architectures and extend the framework to other membrane-protein systems, underscoring the broader utility of QCM-D/electrochemistry correlations for engineering biocatalytic interfaces.
